# Patient experience of communication consistency amongst staff is related to nurse–physician teamwork in hospitals

**DOI:** 10.1002/nop2.431

**Published:** 2020-01-15

**Authors:** Mia von Knorring, Peter Griffiths, Jane Ball, Sara Runesdotter, Rikard Lindqvist

**Affiliations:** ^1^ Department of Learning, Informatics, Management, and Ethics (LIME) Medical Management Center Karolinska Institutet Stockholm Sweden; ^2^ National Institute for Health Research Collaboration for Leadership in Health Research & Care (Wessex) University of Southampton Southampton UK; ^3^ Department of Learning, Informatics, Management, and Ethics (LIME) Division of Innovative Care Research Karolinska Institutet Stockholm Sweden; ^4^ School of Health Sciences University of Southampton Southampton UK; ^5^ Stockholm County Council Stockholm Health Care Services Karolinska Institutet Stockholm Sweden

**Keywords:** communication consistency, England, hospitals, nurse‐physician teamwork, patient experience, RN4CAST

## Abstract

**Aim:**

To investigate whether nurse reported teamwork with physicians was associated with patient perceived consistency in staff‐to‐patient communication.

**Design:**

A cross‐sectional survey design was used, drawing on data collected from two surveys in England.

**Methods:**

Teamwork was assessed using data from the RN4CAST survey of 2,990 nurses in 31 Trusts in England. Data on patient experience derived from the National Health Services Adult Inpatient Questionnaire, including 12,506 patients in the same Trusts. A cross‐sectional design with multivariate logistic regression was used.

**Results:**

Each 5% increase in the proportion of nurses who agree that there “is a lot of teamwork between nurses and physicians” was associated with 7% lower odds that patients reported inconsistency in communication amongst staff. The results suggest that patients seem to experience the consequences of less teamwork between nurses and physicians through their own perceptions of inconsistency in communication between staff. The findings emphasize good teamwork between doctors and nurses are not only important for the team, but also can have consequences for patients. It provides additional incentive to find mechanisms to breakdown disciplinary barriers and improve the cohesion of clinical teams for the benefit of their patients.

## 1 INTRODUCTION

The need for effective teamwork and improved communication amongst caregivers is increasingly recognized in healthcare policy worldwide (International, 2017; Manser, 2009; West & Lyubovnikova, 2013). As healthcare organizations navigate in highly complex contexts, they are largely dependent on thorough collaboration and sharing of information between staff at all levels (West & Lyubovnikova, 2013). Promoting high‐quality teamwork based on effective and frequent communication is therefore essential for developing well‐functioning healthcare organizations (Hughes, 2008; Tang, Zhou, Chan, & Liaw, 2018).

High‐quality teamwork and communication between staff have also been suggested as key factors to assure patient safety (Manser, 2009). Teamwork between nurses and physicians has long been identified as significant for nurse well‐being and nurse‐assessed quality of care (Rafferty, Ball, & Aiken, 2001). Nurse–physician collaboration has also shown to be a positive attribute of the work environment in so‐called magnet hospitals (Lake, 2007; Laschinger & Leiter, 2006), and during the last two decades, an increasing body of research evidence has shown that quality of teamwork is important not only for staff well‐being but also for patient outcomes (Lyubovnikova, West, Dawson, & Carter, 2015; Reason, 1995; West, 2001; West & Lyubovnikova, 2013). A recent study building on data from 62,733 respondents in 147 acute hospitals in the English National Health Service (NHS), Lyubovnikova et al. (2015), showed that prevalence of high‐quality teamwork or real team membership (i.e. where teams build on shared objectives, structural interdependency and regular reflective learning over work to keep track of the overarching objectives) was associated with patient outcome both on individual and organizational levels. Individual staff who reported real team membership witnessed fewer errors and incidents at their workplaces. Hospitals where a larger proportion of staff reported that they worked in real teams also had lower patient mortality rates (i.e. unexpected deaths) (Lyubovnikova et al., 2015).

One aspect of good teamwork is good communication between team members. This, in turn, has potential benefits for patients. According to different studies, between 22%–65% of all severe adverse events are due to or involve communication failures between staff (De Meester, Verspuy, Monsieurs, & Van Bogaert, 2013; Manser, 2009; Martin, Ummenhofer, Manser, & Spirig, 2010; Rabøl et al., 2011). Other reported patient benefits of staff teamwork are fewer physician visits, reduced hospitalization rates and greater satisfaction with care (West & Lyubovnikova, 2013).

Taken together, a large body of research indicate that hospitals with well‐functioning teams that build on collaboration and communication between staff members seem to have better health outcomes for their patients. However, no study has yet explored what impact staff teamwork might have on patients' perceptions of consistency in communication from staff. If communication between team members is a key aspect of a well‐functioning team, one consequence of its absence is that information is not shared and plans not agreed. This, in turn, might have a direct impact on patient experience.

The aim of this study was to investigate whether nurse reported teamwork with physicians was associated to patient perceived consistency in staff‐to‐patient communication.

## 2 METHODS

A cross‐sectional survey design was used, drawing on data collected from two surveys in England. Teamwork between nurses and physicians was assessed using data from the RN4CAST survey of Registered Nurses (RN) undertaken in 2010 (Sermeus et al., 2011). In England, the questionnaire was distributed to a representatively selected sample (based on size, geographic location and teaching status) of 31 Trusts (i.e. governing bodies that consist of conglomerates of hospitals). In each Trust, a stratified random sample of maximum ten medical and surgical wards (five of each) was selected in each hospital. In total, 7,609 registered nurses in the 31 Trusts (covering 46 hospitals and 401 wards) were invited to take part in the study of which 2,990 (39%) responded. The nurse response rate varied between the 31 Trusts from 19%–69%. Nurses reported on the extent to which teamwork between nurses and doctors was present in their current job by responding to one item in the Practice Environment Scale of the Nursing Work Index (PES‐NWI) instrument (Lake, 2002; Li et al., 2007) specifically addressing nurse–physician teamwork.

Data on patient experience were collected from the 2010 National Health Services Adult Inpatient Questionnaire (Care Quality Commission, 2011). It was distributed to 136,460 patients from all 161 acute and specialist Trusts in England. The response rate was 49 per cent. The patients, discharged between June and August 2010, were chosen through purposive selection where each Trust identified a list of a maximum of 850 consequently discharged patients. This study builds on answers from the 12,506 patients who were in the same 31 Trusts as the English RN4CAST survey.

### 2.1 Research Ethics Committee approval

The overall RN4CAST project obtained Research Ethics Committee approval from the ethics committee at Katholieke Universiteit Leuven in Belgium (Ref: B3222009 6682), since the project was coordinated by the researcher from Leuven. Research Ethics Committee approval to undertake the study in England was provided by the National Research Ethics Service (NHS REC ref 09/H0808/69).

Data from the adult inpatient questionnaire are handled in accordance with the standards specified in the Microdata Handling and Security: Guide to Good Practice (https://data-archive.ac.uk/media/132701/UKDA171-SS-MicrodataHandling.pdf).

### 2.2 Variables

#### 2.2.1 Explanatory variables

In the RN survey participants were asked to grade if they agreed to several statements related to their work environment. One of these statements directly addressed the issue of teamwork: “A lot of teamwork between nurses and physicians.” The response in the survey where given on a 4‐point Likert scale ranging from 1 = strongly disagree to 4 = strongly agree. The variable was dichotomized into the positive responses (3 = somewhat agree and 4 = strongly agree) versus negative response (1 = strongly disagree and 2 = somewhat disagree) (see Table 1). Since the analysis was made on Trust level, we calculated the proportion of nurses reporting a positive response.

**Table 1:** Response patterns of patient and nurses at Trust level

	Overall proportion in per cent	Lowest proportion on Trust level in per cent	Highest proportion on Trust level in per cent
**Responses of Patients (in Trust)**: Sometimes in a hospital, a member of staff will say one thing and another will say something quite different. Did this happen to you?			
Yes, often/Yes, sometimes	36%	30%	48%
No	64%	52%	70%
**Responses of Nurses (in Trust):** Please indicate the extent to which you agree that each of the following features is present in your current job. A lot of teamwork between nurses and physicians			
Somewhat Agree/Strongly Agree	77%	66%	90%
Strongly Disagree/Somewhat Disagree	23%	10%	34%

From the patient experiences survey, we selected the question: “Sometimes in a hospital, a member of staff will say one thing and another will say something quite different. Did this happen to you?” The answers were given in three alternatives: “yes, often,” “yes, sometimes” and “no”. The measure was dichotomized as either patients agreeing to the statement (“yes, often”/”yes, sometimes”) or disagreeing (“no”).

#### 2.2.2 Control variables

Four variables were selected as control variables. For the nurses, gender (proportion female) and work experience as a nurse (mean number of years) were used. Using nurses' age as control variable was considered but excluded due to a high correlation between age and work experience. In the patient experience data, gender and age (66 or older) were controlled for.

### 2.3 Statistical methods

Descriptive statistics, cross‐tabulations, frequencies and graphs depicting distributions and correlations were used to check for anomalies, such as outliers or extreme values. We used adjusted multivariate logistic regression models to estimate the relationship of RN‐assessed teamwork and patients' experience of mixed messages from staff. A mixed model was used to correct for the dependency of observations in Trusts. Confidence intervals (CI) were set at 95%. Data were analysed using SAS 9.4.

## 3 RESULTS

The 31 Trusts included in these analyses consist of 46 hospitals. The respondents consist of 12,260 patients and 2,919 nurses (71 of the nurses were not included due to missing data on the relevant variables). The mean age of nurses by Trust ranged between 34–46 years and the proportion of women varied between 78%–99%. The average work experience in years on Trust level varied between a minimum of 8 and a maximum of 19. The range of older patients (66+) by Trust was 39%–62%, and the proportion of female patients varied between 46%–62%.

77% of the nurses responded that they somewhat or strongly agreed to the statement that there was a lot of teamwork between nurses and physicians at their current workplace, ranging by Trust between 66%–90%. A little more than one third (36%) of the patients responded that they had sometimes or often experienced that a member of staff said one thing and that another said something quite different. The proportion ranged by Trust between 30%–48% (Table 1).

The results of the unadjusted and adjusted model of Trust level nurse assessments of teamwork between nurses and physicians and their relationship with patient perceptions of inconsistent communication from staff are presented in Table 2. Gender of nurses and patients and patient age were significantly associated with the proportion of patients who perceived inconsistent communication from staff. Years of work experience amongst nursing staff was not significantly related to patient perception of communication.

**Table 2:** Effect of nurses experience of teamwork on patients perceived inconsistency in communication amongst staff

	Bivariate analyses		Pr > |*t*|	Multivariate analysis^a^		Pr > |*t*|
	Odds ratio	95%CI (Low, High)		Odds ratio	95%CI (Low, High)	
Nurses agreeing in the statement "A lot of teamwork between nurses and physicians"	0.986	0.977, 0.995	0.0017	0.985	0.977, 0.994	0.0011
Nurses mean years of work experience	1.022	0.997, 1.047	0.0893	1.020	0.998, 1.043	0.0777
Gender of nurses (proportion of female nurses)	0.375	0.112, 1.253	0.1112	0.294	0.104, 0.833	0.0212
Age of patients (66> years)	0.607	0.563, 0.654	<0.0001	0.619	0.575, 0.668	<0.0001
Gender of patients (female)	1.311	1.217, 1.413	<0.0001	1.265	1.173, 1.364	<0.0001
Error variance						
Level‐2 intercept “Trust”				0.0081		
Odds ratio of a 5% increase of the proportion of nurses agreeing on the statement "A lot of teamwork between nurses and physicians"	0.932	0.890, 0.975	0.0017	0.927	0.890, 970	0.0011

a Adjusted for patients age 66 years>, gender for patients, years of experience for nurses, gender for nurses.

In the adjusted model, each 5% increase in the proportion of nurses who agree to the statement that there “is a lot of teamwork between nurses and physicians” is associated with 7% lower odds that the patients experience inconsistency in communication amongst staff.

## 4 DISCUSSION

There is considerable variation between Trusts in the extent that teamwork is reported by nurses and that inconsistent communication is reported by patients. After controlling for other factors, variation in nurse reported teamwork is found to be significantly associated with patient experience of inconsistent communication; a five per cent increase in the proportion of nurses reporting good teamwork between nurses and physicians is associated with a seven per cent decrease in the odds of patients getting inconsistent information from staff. This finding establishes a link between staff teamwork and consistency in communication to patients.

Both from an organizational and patient perspective, this finding has clinical relevance, given that the proportion of nurses reporting poor teamwork varies from 10–34 per cent. That we can see such an effect at the Trust level could be seen as surprising, given that NHS Trust is the highest organizational level comprising a large number of different organizational settings, so differences at hospitals and ward level are masked. Another finding is that a measure of patient experience not only provides an indication of care quality, as shown by Aiken et al., 2012 (Aiken et al., 2012), but also provides an indicator of teamwork.

Our findings support the findings in other studies that quality of teamwork has consequences for patients (Lyubovnikova et al., 2015; Reason, 1995; West, 2001; West & Lyubovnikova, 2013). What this study adds is that inconsistency in communication from staff to patients might be a consequence of lack of teamwork. This may put patients at risk since other studies have found that conflicting information to the patient has effect on patient safety (Manser, 2009). Inconsistent communication to patients may potentially also erode patient confidence for healthcare staff and, in the long run, potentially for the healthcare system at large.

Our study has limitations. Patients were asked about consistency in communication between staff in general and not nurses and doctors specifically. However, nurses and physicians represent the two largest categories of staff interacting with patients (Page, 2004). Furthermore, even in cases where nurses might have been the ones given conflicting information to the patient, we would argue that good teamwork between nurses and physicians probably is rare unless there is satisfactory collaboration amongst the group of nurses at the work place. The fact that the specific link between the nurses answering the survey and patients responding is unknown, beyond the fact that nurses worked on wards in the hospital where the patients spent their inpatient stay can be considered as a limitation. However, a large and significant body of work including (for example) Aiken et al's seminal paper in the Lancet (Aiken et al., 2014) on the association between hospital ward staffing levels and mortality uses similar methodology. Because of this loose linkage, it is possible that the association we observe is may be an under or overestimate of the true relationship.

Another limitation is that the concept teamwork was not defined in the questionnaire. Therefore, we cannot say anything about type and quality of teamwork the nurses referred to. West and colleagues have emphasized the need for a clear differentiation between actual work in what they call “real teams” and “pseudo teams” (Lyubovnikova et al., 2015; West & Lyubovnikova, 2013). Given the data used in this study, we do not know to what extent the nurses referred to real teams or pseudo teams in their answers. We can only state that there is a significant correlation between what nurses perceive as a team and patients' perceptions of consistency in information from staff.

Further studies are needed to explore potential impact of differences in type of teams.

The main result was not affected by gender, age or experience amongst the nurses. Given the small number of observations (e.g. Trusts), the possibility of controlling for more items in the model was limited. Therefore, we cannot exclude the possibility that other intermediate factors might have affected the result.

Whilst this remains a cross‐sectional study with inherent limitations in inferring cause, a key strength of this study is that we are not correlating variables in a single survey and so common method bias cannot account for our findings. The data used here are collected in 2010 can be considered as old, however, whilst levels of the reported variables may well have changed over time it seems less probably that the relationship would. And even though the data used are from 2010, it is still the only comprehensive data available on nurse perceptions and patient experience covering several Trusts in England (Aiken et al., 2018).

## 5 CONCLUSIONS

What this study adds is showing a relationship that indicates that teamwork has consequences for the patients even when measured at high organizational levels in healthcare systems. Patients actually appear to be experiencing the consequences of less teamwork through their own perceptions of inconsistency in communication between staff.

## 6 IMPLICATIONS FOR NURSING MANAGEMENT

The findings emphasize the importance of good teamwork between doctors and nurses. It provides additional incentive to find mechanisms to breakdown disciplinary barriers and improve the cohesion of clinical teams for the benefit of their patients.

## CONFLICT OF INTEREST

Authors declare no conflict of interest.
